# Interleukin 33 Selectively Augments Rhinovirus-Induced Type 2 Immune Responses in Asthmatic but not Healthy People

**DOI:** 10.3389/fimmu.2018.01895

**Published:** 2018-08-16

**Authors:** Lisa M. Jurak, Yang Xi, Megan Landgraf, Melanie L. Carroll, Liisa Murray, John W. Upham

**Affiliations:** ^1^Lung and Allergy Research Centre, Diamantina Institute, University of Queensland, Woolloongabba, QLD, Australia; ^2^Department of Respiratory Medicine, Princess Alexandra Hospital, Brisbane, QLD, Australia

**Keywords:** asthma, IL-33, rhinovirus, type 2 immunity, ST2, innate lymphoid cells

## Abstract

Interleukin- 33 (IL-33) is an epithelial-derived cytokine that initiates type 2 immune responses to allergens, though whether IL-33 has the ability to modify responses to respiratory viral infections remains unclear. This study aimed to investigate the effects of IL-33 on rhinovirus (RV)-induced immune responses by circulating leukocytes from people with allergic asthma, and how this response may differ from non-allergic controls. Our experimental approach involved co-exposing peripheral blood mononuclear cells to IL-33 and RV in order to model how the functions of virus-responsive lymphocytes could be modified after recruitment to an airway environment enriched in IL-33. In the current study, IL-33 enhanced RV-induced IL-5 and IL-13 release by cells from people with allergic asthma, but had no effect on IL-5 and IL-13 production by cells from healthy donors. In asthmatic individuals, IL-33 also enhanced mRNA and surface protein expression of ST2 (the IL-33 receptor IL1RL1), while soluble ST2 concentrations were low. In contrast, IL-33 had no effect on mRNA and surface expression of ST2 in healthy individuals. In people with allergic asthma, RV-activated ST2^+^ innate lymphoid cells (ST2^+^ILC) were the predominant source of IL-33 augmented IL-13 release. In contrast, RV-activated natural killer cells (NK cells) were the predominant source of IL-33 augmented IFNγ release in healthy individuals. This suggests that the effects of IL-33 on the cellular immune response to RV differ between asthmatic and healthy individuals. These findings provide a mechanism by which RV infections and IL-33 might interact in asthmatic individuals to exacerbate type 2 immune responses and allergic airway inflammation.

## Introduction

Acute exacerbations make a major contribution to the burden of asthma, with respiratory viral infections, in particular, rhinoviruses (RV), triggering most exacerbations. However, the underlying mechanisms by which RV trigger serious asthma exacerbations are not well understood. Even though RV is usually confined to epithelial cells lining the upper respiratory tract and may sometimes involve the lower respiratory tract, there is strong evidence that systemic immune function plays an important role in host defense against RV infections. High titers of specific neutralizing antibodies are protective against experimental RV infections (1). Moreover, the capacity of circulating T-cells to proliferate and produce IFNγ and IL-10 after RV stimulation *in vitro* is inversely associated with viral load during an RV infection (2). More recently, a large birth cohort study has shown that RV-activated blood mononuclear cells exhibit varying cytokine response patterns *in vitro* that are associated with different clinical outcomes (3).

Viruses typically trigger a type 1 (Th1) immune response in most individuals, yet, they are a common trigger of asthma exacerbations, a disease in which type 2 (Th2) immune responses are often prominent, with type 2 innate lymphoid cells (ILC2), allergen activated Th2 cells, mast cells, and eosinophils all playing key roles. Increased capacity for type 2 cytokine production in asthmatic individuals is linked to more severe virus-induced asthma symptoms and airway eosinophilia following experimental RV challenge (4). Moreover, immune responses to RV in asthma are somewhat Th1 deficient and may be skewed toward a Th2 response (5, 6).

Interleukin- 33 (IL-33) is a strong inducer of Th2 immune responses. Recent studies in mice and humans have implicated the importance of IL-33 in the development of Th2 inflammation during RV infection (6, 7), thus providing a possible mechanistic link between viral infections and eosinophilic inflammation. Asthma is associated with variants in the genes encoding IL-33 and its receptor ST2 (also known as ST2L and IL1RL1) (8, 9). The IL-33 receptor itself comprises two proteins ST2 and IL-1RacP. IL-33 binds to ST2 causing a conformational change that enables it to interact with IL-1RacP (10). Epithelial cells and macrophages release IL-33, which activate Th2 cells, ILC2 cells, and other immune cells *via* ST2, thereby amplifying type 2 immune responses (11). However, ST2 can also exist as a soluble receptor (sST2), which has the ability to neutralize the effects of IL-33 (12). Although, viral infections such as influenza can induce IL-33 release in murine lungs (13), it is not clear whether IL-33 can modify cellular responses directed against respiratory viruses.

A study of experimental RV infection in asthma showed that virus-induced IL-33 release correlated with high IL-5 and IL-13 concentrations in the airway (6). However, RV infection induced similar IL-33 release in asthmatic and control subjects, suggesting that virus-induced IL-33 release *per se* may not be sufficient to discriminate between asthmatic and healthy responses to RV (6). More recently, Werder et al. (14) showed that during RV infection of mice with allergic airways disease, RV has the ability to amplify inflammation through IL-33 (14). Whether IL-33 can modify cellular responses to respiratory viruses in humans is unknown.

Thus, the objective of the current study was to examine the effects of IL-33 on RV-induced cellular immune responses *in vitro* and to determine whether this differs in asthmatic and healthy individuals. Our approach sought to model how virus-responsive lymphocyte function could be modified after recruitment to an airway environment enriched in IL-33.

## Materials and Methods

### Patients

The Metro South Human Research Ethics Committee approved this study (Ethics approval number 2008000037) and written informed consent was obtained from all participants. A detail questionnaire documented respiratory symptoms, prior diagnoses, and medication use. Healthy subjects had no symptoms or prior diagnosis of respiratory disease. Asthma diagnoses had been confirmed by a physician, and symptoms had occurred within the last 12 months. Current smokers and those with respiratory infection within the preceding 4 weeks were excluded. Allergic sensitization was assessed by skin prick test using a panel of common aeroallergens (grass pollens, house dust mite, domestic animals, and molds). Those asthmatics taking inhaled corticosteroids were asked to withhold these for 24 h prior to blood collection.

### Isolation of Peripheral Blood Mononuclear Cells (PBMCs)

Peripheral blood mononuclear cells were isolated from venous blood and cultured as described (15). PBMCs were exposed to recombinant IL-33 (Preprotech) or media for 6 h prior to addition of RV strain 16 (multiplicity of infection = 1). Cultures were incubated at 37°C with 5% CO_2_ for 5 days (a time dominated by antigen specific recall responses). On the basis of initial dose response experiments, IL-33 was used at 10 ng/mL for all subsequent experiments.

### Quantitative Real-Time PCR

Total RNA was extracted from cell pellets obtained from 5-day cultures, using RNeasy micro RNA kit (Qiagen, Hilden, Germany). The ratio of absorbance at 260 and 280 nm of RNA was measured using Nanodrop (Thermo Scientific, Waltham, MA, USA). RNA was immediately converted to complimentary DNA (cDNA) using cDNA synthesis Kit (Bioline, Alexandria, NSW, Australia) and cycling conditions as per the manufactures instructions. Quantitative real-time polymerase chain reaction (qRT-PCR) contained three volumes of SYBER green master mix (Bioline, Alexandria, NSW, Australia), two volumes of cDNA, and 0.6 volumes of standard primers specific for membrane bound ST2 (Forward primer: 5′-*GGAAAAAACGCAAACCTA-*3′, Reverse primer: 5′*-GGCCTCAATCCAGAACATTTT-*3′) and IL-1RAcP (Forward primer: 5′-*CTGAGGATCTCAAGCGCAGCTA-*3′, Reverse primer: 5′-*AGCAGGACTGTGGCTCCAAAAC-*3′) as indicated. The qRT-PCR cycle consisted of an initial incubation at 95°C for 2 min followed by a subsequent 40 cycles of denaturation and annealing at 95°C for 5 s and 60°C for 10 s. Each reaction was conducted in duplicate, using a Roche light cycler 480 real-time PCR system (Roche, North Ryde, Australia). Analysis was conducted using light cycler 480 software 1.5.0. Genes of interest were calculated in relation to the reference gene glyceraldehyde 3-phosphate dehydrogenase (GAPDH) using the delta–delta Ct method. Gene expression was normalized to a corresponding control sample for each donor.

### ELISA

Cell free supernatants were collected and stored at −20°C until required. The concentration of IL-5, IL 13, IFNγ (BD Biosciences, Franklin Lakes, NJ, USA), and soluble ST2 (R&D systems, Minneapolis, MN, USA) were performed using commercially available ELISA kits, according to the manufacturer’s instructions. Results are presented as amount of cytokine in cell-free supernatants. For soluble ST2 ELISA, the limit of detection for the assay was 31.3 pg/mL. In samples in which the concentration was below the lower limit of detection, it was not possible to determine whether the true value was 0, so, the concentration was arbitrarily assigned to be half the lower limit of detection.

### Flow Cytometry

In this study, flow cytometry was used to evaluate (1) the expression of ST2 on ILCs and T-cells; and (2) the effect of IL-33 on RV-activated immune cells (ST2^+^ ILCs, T cells, and NK cells) and the production of type 1 and type 2 cytokines in human PBMC. For cells undergoing intracellular cytokine staining, cells were incubated in the presence of Brefedlin A for 4 h prior to staining. Cell pellets were then harvested from 5-day cultures and washed twice with PBS and labeled with live/dead (L/D) aqua viability dye (Thermo Scientific, Waltham, MA, USA) for discrimination of dead cells. Cells were then stained with the following combinations of surface antibodies: (1) Lin-1-FITC (CD3/CD14/CD16/CD19/CD20/CD56), CD45-APC-H7, CD127-PEcy7, and ST2-PE; (2) CD3-APCcy7, CD4-FITC; or (3) CD56-AF488, for 30 min on ice and protected from light. Following surface staining, cells were then fixed and permeabilized using Fixation/Permeabilization solution kit (BD bioscience cat#554715). Cells were then intracellularly stained with IL-13-BV421 and IFNγ-PerCPcy5.5 for 30 min at room temperature, protected from light. Cells were fixed in 2% paraformaldehyde. Data were acquired using an LSRFortessa X-20 (BD Bioscience, San Jose, CA, USA) collecting 200,000 total events. Data were analyzed using FlowJo Tree star software (Version 7.6.1). Cell types have been defined as following: T cells (CD3^+^ CD4^+^), and NK cells (CD56^+^). In some experiments, NK cells were further defined as CD56^+^, CD3 negative. ST2^+^ILCs were defined as LIN1^−^FcεR1^−^CD45^+^ CD127^+^ ST2^+^ as previously described (16, 17). Information about the gating strategy and antibodies used in this work have been described in Figure S1 and Table S1 in Supplementary Material.

### Statistical Analysis

Results were analyzed in Graphpad prism using ANOVA with Bonferroni correction, together with two-way comparisons used paired and unpaired *t*-tests where appropriate. Data are shown as mean ± SEM. Differences were regarded as statistically significant at *p* ≤ 0.05.

## Results

### Clinical Characteristics

Participants were between 18 and 53 years of age and comprised 18 people with mild/moderate asthma and 22 non-asthmatic volunteers. Based on skin-prick testing, people with asthma had a greater prevalence of allergic sensitization than non-asthmatic donors (66 versus 36%, respectively), though this difference was not statistically significant as determined by χ^2^ analysis. Serum total IgE concentrations were significantly higher in the asthmatic group than in the non-asthmatic group (mean values = 361 and 194 IU/L, respectively; *p* < 0.0125).

### Effects of IL-33 Pre-Exposure on RV-Induced Type 1 and 2 Cytokine Release

**Figure 1:**
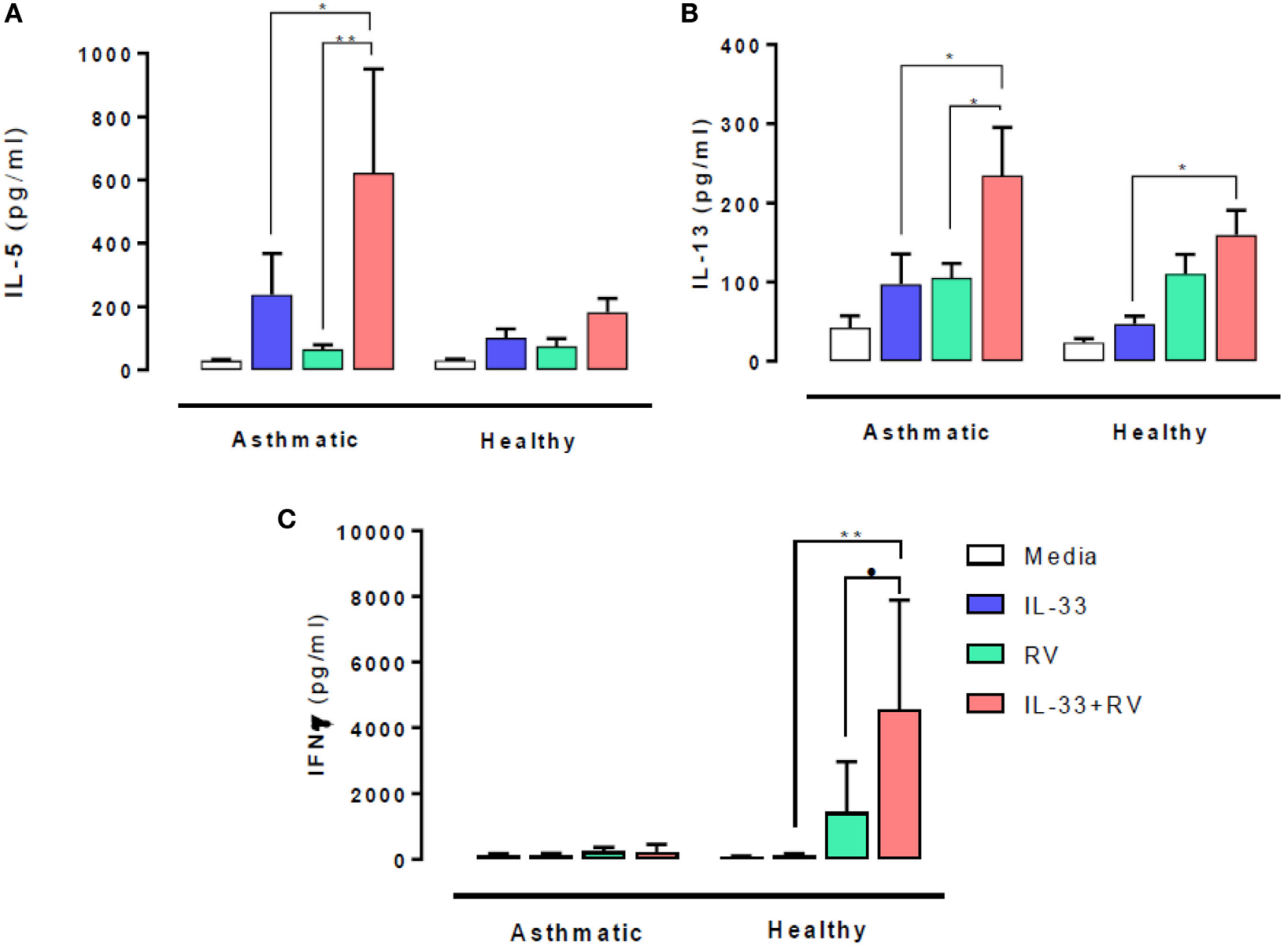
Effects of IL-33 on rhinovirus (RV)-induced type 1 and 2 cytokine release. PBMCs from people with allergic asthma (*n* = 14) and non-allergic healthy controls (*n* = 22) were pre-exposed to media or IL-33 for 6 h and then cultured in the absence or presence of RV for 5 days. IL-5 **(A)**, IL-13 **(B)**, and IFNγ **(C)** production have been measured by ELISA. Data are expressed as mean (±SEM). Abbreviations: PBMC, peripheral blood mononuclear cells; IL-33, interleukin 33; RV, rhinovirus 16; ns, not significant. *p < 0.05, **p < 0.01, ***p < 0.001.

The effect of IL-33 on RV-induced type 1 and 2 cytokine release was evaluated 5 days post stimulation. In people with asthma, IL-33 pre-exposure led to significantly higher RV-induced (in red) IL-5 (Figure 1A) and IL-13 (Figure 1B) release (in red) compared to RV alone (in green) (*p* = 0.02 and *p* = 0.003 respectively). IL-33 alone (in blue) also induced IL-13 release in asthmatic individuals, which increased further in the presence of RV. Though IL-5 and IL-13 secretion tended to be higher in those with asthma than in healthy subjects, these differences were not statistically significant. In contrast, IL-33 pre-exposure had no significant effects on RV-induced IL-5 and IL-13 release by cells from healthy donors.

IFNγ release at day 5 post RV stimulation showed a very different response pattern (Figure 1C). RV-induced IFNγ release was lower in cells from asthmatics than in healthy donors (*p* = 0.03) and was not altered by IL-33 pre-exposure. In contrast, IL-33 pre-exposure led to significantly higher RV-induced IFNγ release by cells from healthy donors (*p* = 0.001).

### Effects of IL-33 on Membrane-Bound ST2 (IL1-RL1) and Soluble ST2 (sST2) in Asthmatic and Healthy Individuals

**Figure 2:**
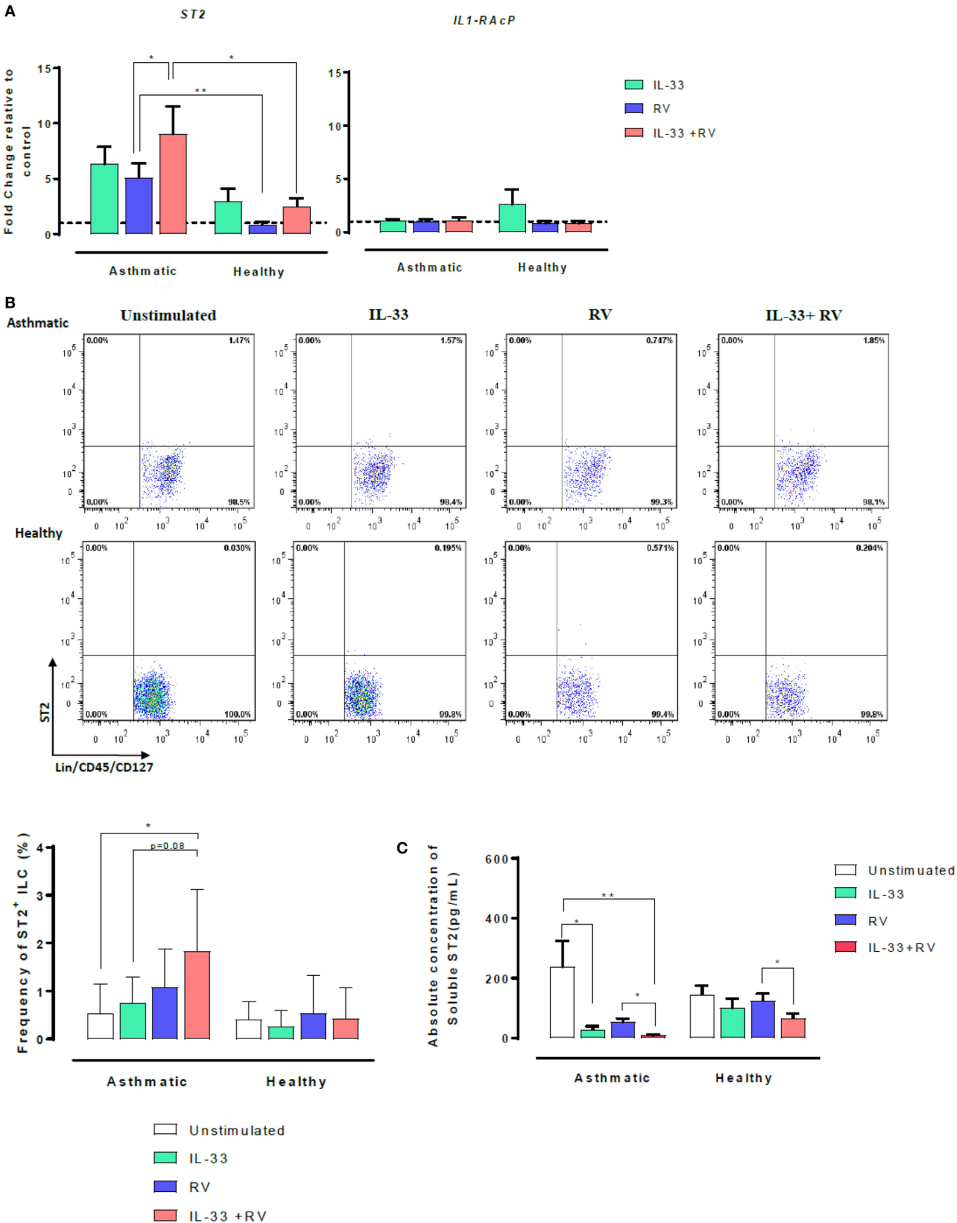
Effects of IL-33 on membrane-bound ST2 and soluble ST2 (sST2) in asthmatic and healthy individuals. PBMCs from patients with allergic asthma (*n* = 12) or non-allergic healthy controls (*n* = 16) were exposed to IL-33 or media for 6 h and then cultured in the absence or presence of RV for 5 days. **(A)** Relative expression of *ST2* and *IL-1RAcP* was assessed by qRT-PCR using primer pairs corresponding to each receptor and normalized to the house-keeping gene GAPDH. Basal expression is represented by a broken line. **(B)** The frequency of ST2L^+^ ILC were then assessed by flow cytometry. ILCs were defined as Lin^−^FcεR1^−^CD45^+^CD127^+^ and then assessed for ST2. Raw dot plots representative of ST2^+^ ILC from asthmatic and healthy donors. Data are presented as mean (±SEM) from 8 asthmatic and 6 healthy donors. **(C)** Soluble ST2 concentration was determined from cells free supernatants by ELISA. Data are expressed as mean (±SEM) from duplicate measurements. Abbreviations: PBMC, peripheral blood mononuclear cells; IL-33, interleukin 33; RV, rhinovirus 16; ST2^+^ ILC2, ST2^+^ innate lymphoid cells; qRT-PCR, quantitative real-time polymerase chain reaction; GAPDH, glyceraldehyde 3-phosphate dehydrogenase. *p < 0.05, **p < 0.01.

The IL-33 membrane-bound receptor comprises two proteins, ST2 and its accessory receptor IL-1RAcP. However, ST2 can also exist as a soluble form named sST2. We, therefore, sought to determine whether IL-33 modulates ST2 and IL-1RAcP on RV-activated cells. Expression of *ST2* and *IL-1RAcP* were assessed using qRT-PCR (Figure 2A). Overall, cellular *ST2* expression was higher in asthmatic than healthy individuals (Figure 2A, left). RV-induced *ST2* expression was significantly higher in cells from asthmatic donors than in cells from healthy donors (in blue). Pre-exposure to IL-33 followed by RV (IL-33 + RV) (in red) further augmented the difference in *ST2* expression between asthmatic and healthy donors. In cells from asthmatics, IL-33 + RV stimulation lead to a ninefold increase in *ST2* expression, which was significantly higher than cells exposed to RV alone. In cells from healthy donors, RV and IL-33 (alone and in combination) had no significant effects on *ST2* expression. In contrast, *IL-1RAcP* expression was similar in cells from asthmatic and healthy individuals. Similarly, RV and IL-33 did not modify *IL-1RAcP* expression (Figure 2A, right).

To further investigate the increased *ST2* mRNA expression in asthmatic cells stimulated with IL-33 + RV, cell surface ST2 protein expression was assessed using flow cytometry, with a combination of surface antigens specific for innate lymphoid cells (ILC) (Figure 2B) and conventional T-cells (Figure S3 in Supplementary Material). Consistent with our qRT-PCR data, the frequency of ST2^+^ILC was generally higher in asthmatic donors in comparison to healthy donors when stimulated under the same conditions (Figure 2, left). In asthmatic donors, IL-33 + RV caused a greater increase in the frequency of ST2^+^ILC than RV and IL-33 individually (Figure 2B, right). In healthy donors, IL-33, RV, and IL-33 + RV had no effect on ST2^+^ ILC. Only a small proportion of unstimulated ILCs and T-cells were ST2^+^, and there was no significant difference between cells from asthmatic and healthy donors (Figure 2B, Figure S3 in Supplementary Material). IL-33, RV, and IL-33 + RV had no appreciable effect on the proportion of ST2^+^ expressing T cells (Figure S3 in Supplementary Material).

Soluble ST2 (sST2) concentrations were measured from cell-free supernatants from 5-day PBMC cultures by ELISA (Figure 2C). In asthmatic individuals, the concentration of sST2 was significantly lower in IL-33 and IL-33 + RV stimulated cells than in unstimulated cells. There was a trend for lower sST2 concentrations with RV alone, but this was not statistically significant (Figure 2C). In contrast, both IL-33 and RV had minimal effects on sST2 concentrations in healthy individuals in comparison to unstimulated cells (Figure 2C). Interestingly, we found that IL-33 pre-exposure can further dampen RV-induced sST2 response, and this was observed in both asthmatic and healthy PBMC.

### Which Cells Are Producing Type 1 and Type 2 Cytokines in Peripheral Blood?

**Figure 3:**
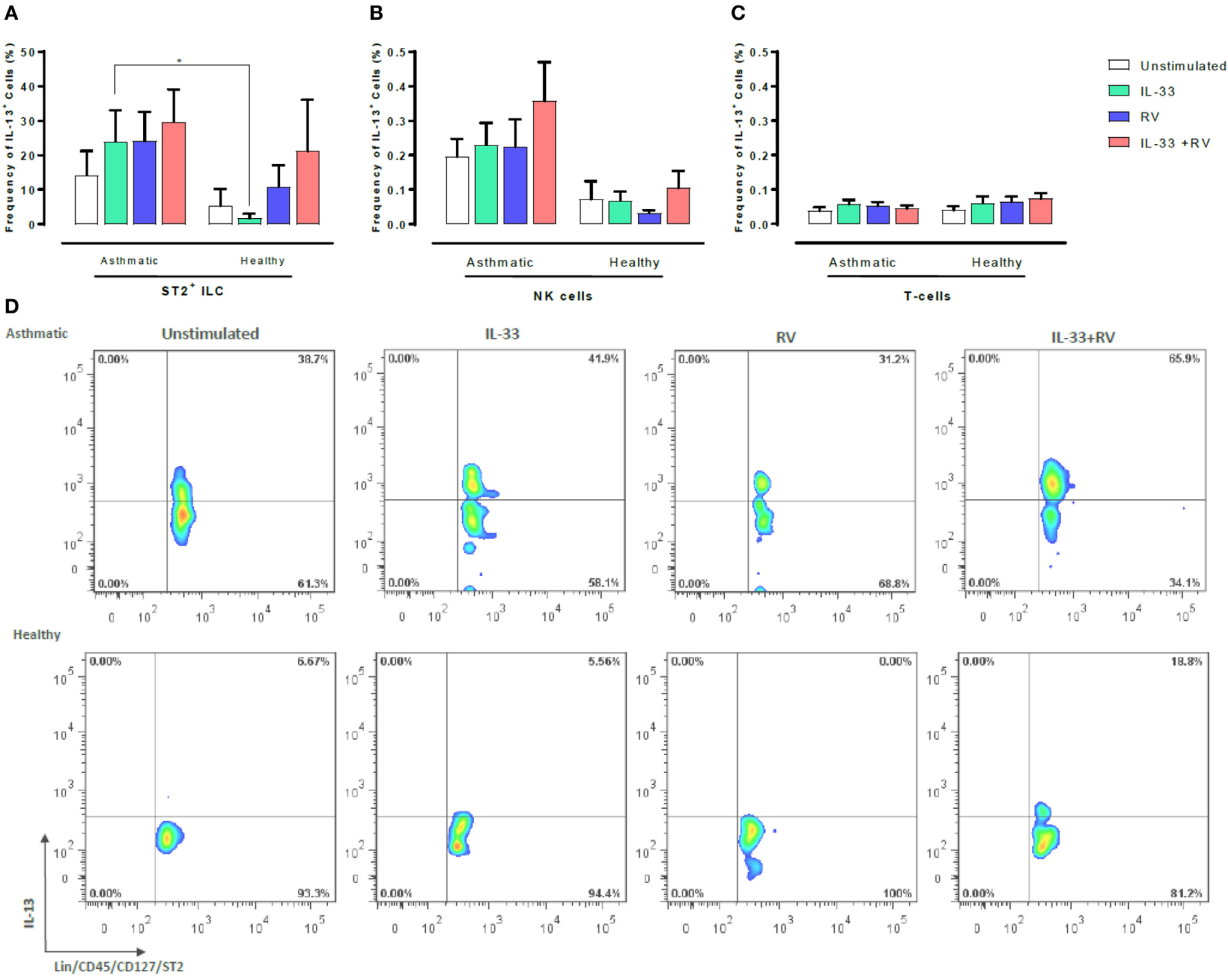
Evaluation of IL-13-producing ST2^+^ILC, CD56^+^, and CD3^+^ T cells in human PBMC. IL-13-producing cell subsets were evaluated using flow cytometry **(A–C)**. Raw dot plots representative of IL-13 + ST2 + ILC from 8 asthmatic and 6 healthy donors. PBMCs from people with allergic asthma and non-allergic healthy controls were pre-exposed to media or IL-33 for 6 h and then cultured in the absence or presence of RV for 5 days **(D)**. Abbreviations: PBMC, peripheral blood mononuclear cells; IL-33, interleukin 33; RV, rhinovirus 16, ST2^+^ILCs, ST2^+^ innate lymphoid cells. *p ≤ 0.05 and **p ≤ 0.01.

**Figure 4:**
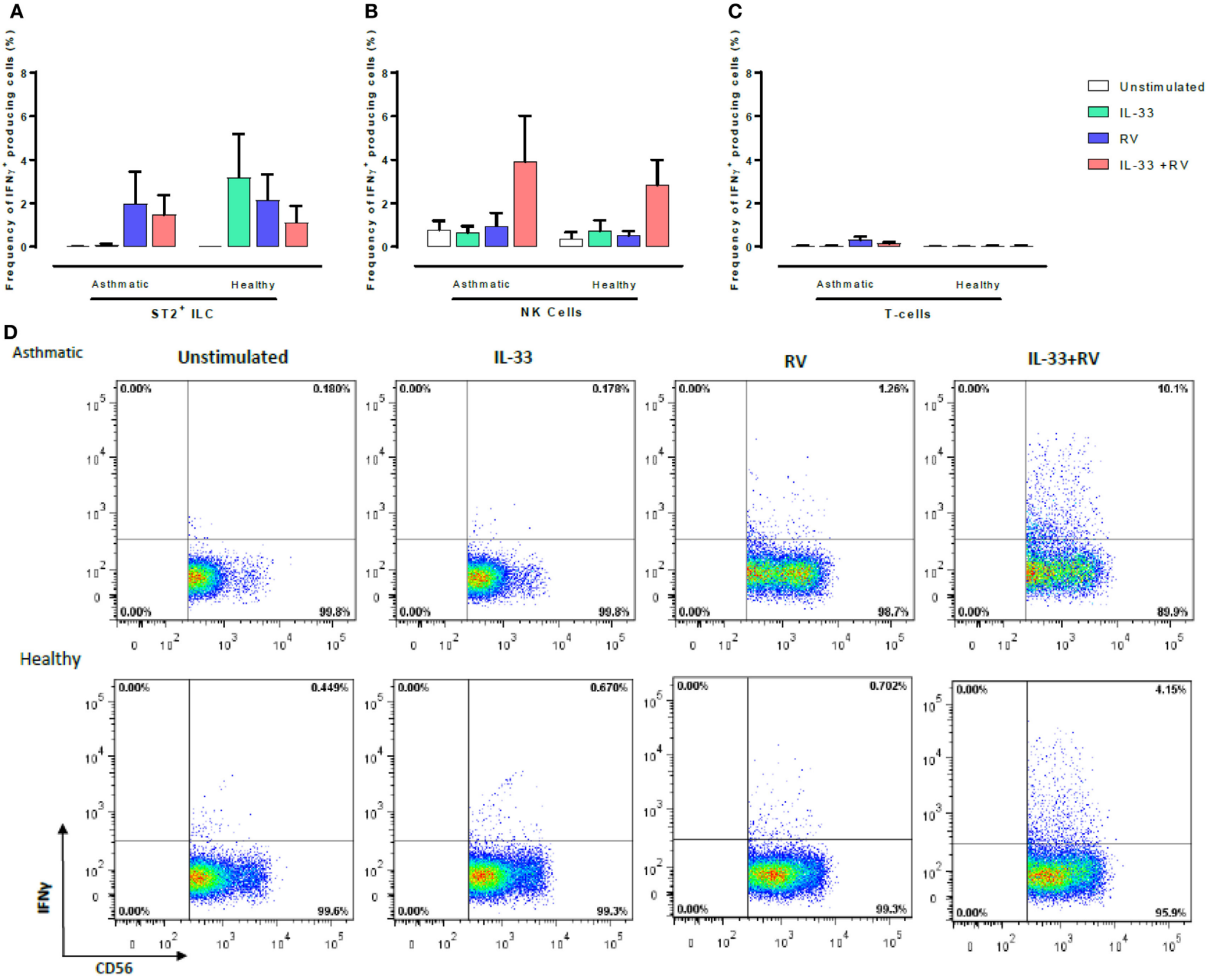
Evaluation of IFNγ-producing ST2^+^ILC, CD56^+^, and CD3^+^ T cells in human PBMC. IFN-γ-producing cell subsets were evaluated using flow cytometry **(A–C)**. Raw dot plots representative of IFN-γ^+^CD56^+^ cells from 8 asthmatic and 6 healthy donors. PBMCs from people with allergic asthma and non-allergic healthy controls were pre-exposed to media or IL-33 for 6 h and then cultured in the absence or presence of RV for 5 days **(D)**. Abbreviations: PBMC, peripheral blood mononuclear cells; IL-33, interleukin 33; RV, rhinovirus 16; ST2^+^ILCs, ST2^+^ innate lymphoid cells. *p ≤ 0.05 and **p ≤ 0.01.

We next sought to determine which leukocyte subsets respond to IL-33 and RV by producing type 1 and type 2 cytokines. Flow cytometry was used to determine the proportion of IL-13 or IFNγ producing ST2^+^ILC, CD56^+^ cells, and T-cells (Figures 3 and 4). ST2^+^ILC were the main IL-13 producers in asthmatic individuals (Figure 3). IL-33 alone significantly increased the frequency of IL-13 producing ST2^+^ ILC in asthmatic individuals in comparison to healthy. There was a trend toward a higher frequency of IL-13 producing ST2^+^ILC in asthmatic donors in comparison to healthy donors when stimulated with RV alone and in combination with IL-33. However, this trend was not significant (Figure 3). In contrast, IL-33 pre-exposure had no significant effect on the frequency of IL-13 producing NK cells or T-cells in asthmatic and healthy individuals (Figure 3).

CD56^+^ cells were the predominant IFNγ-producing cells in both healthy and asthmatic donors (Figure 4), and this was most pronounced in unstimulated samples and those stimulated with IL-33 + RV (Figure 4B). Small numbers of IFNγ producing ST2^+^ILC and T-cells were observed; however, there was little evidence that this was modified by IL-33 or RV (Figure 4). While CD56 can be expressed by both NK cells and conventional T-cells, it seems that the great majority of IFNγ producing CD56^+^ cells (Figure 4; Figure S4 in Supplementary Material) were NK cells rather than CD56^+^ CD3^+^ T-cells, as very few IFNγ-producing CD3^+^ T-cells were identified. Moreover, additional experiments performed in a subset of participants identified that CD56^+^ cells lacking CD3, CD14, and CD19 were the predominant source of IFNγ under the various stimuli used in our experiments (Figure S4 in Supplementary Material).

## Discussion

This study demonstrates that IL-33 has distinct effects on the *in vitro* cellular response to RV in asthmatic and healthy individuals. In asthmatics, IL-33 augments type 2 inflammatory responses to RV, but has little effect on type 1 immune responses. In healthy individuals, IL-33 enhances RV-induced type 1 immune response, but has no effect on type 2 responses. This is associated with differential regulation of the IL-33 receptor, with higher stimulated ST2 expression in cells from asthmatic than in cells from healthy individuals. In contrast, unstimulated ST2 expression and ST2 surface staining was similar in asthmatic and healthy participants. Our findings provide a possible mechanism by which IL-33 release amplifies eosinophilic inflammation in asthmatic individuals, yet has what appears to be a protective role in healthy individuals.

Given that ST2 was initially thought to be restricted to Th2 cells (18), it is surprising that circulating immune cells from both asthmatic and healthy individuals have a similar ability to respond to IL-33. More recent studies have identified that ST2 is more widely expressed on other immune cells (19). New evidence has emerged that Th1 effector cells can transiently express *ST2* during experimental viral infection and that Th1 effector cell differentiation and cytokine production is dependent on the IL-33/ST2 axis (20). This provides a plausible mechanism by which IL-33 was able to augment IFNγ release by RV-activated cells from healthy donors in the current study. Supporting this concept, our gene expression data show that cells from healthy donors express ST2 (Figure 2A).

Previous investigators have examined IL-33 receptor expression in asthma. Traister and colleagues demonstrated high airway epithelial ST2 expression in severe asthma, and importantly, linked ST2 expression to exacerbation risk and markers of type 2 inflammation (21). The IL-33 receptor is highly complex, as the *IL1RL1* gene is comprised of two splice variants that have opposing functional effects. The membrane bound form, ST2, which helps drive Th2 inflammation, and a soluble form sST2, which acts as a decoy receptor and neutralizes the effect of IL-33 (12).

Our study for the first time simultaneously compared the relative balance of membrane bound ST2 and sST2 in asthmatic and healthy individuals, and how this is regulated *in vitro* by IL-33 and RV in the setting of asthma. In those with asthma, IL-33 pre-exposure and RV stimulation augmented *ST2* mRNA expression and elevated the frequency of ST2^+^ILC, whereas sST2 production was low. In healthy subjects, IL-33 had little effect on RV-induced *ST2* mRNA and frequency of membrane bound ST2 on ILCs and T-cells, whereas sST2 was readily detected. This skewed expression of each splice variant may partially explain why IL-33 has the ability to amplify type 2 responses during viral infection in asthmatic individuals while having a protective role in healthy individuals. However, the underlying reason as to why one splice variant is translated over another remains unknown. Genome wide association studies have identified single nucleotide polymorphisms (SNPs) in the *IL1RL1* gene that are associated with asthma (21–23). Furthermore, SNPs located in the promoter region, have been linked to higher transcriptional activity of the *IL1RL1* gene (22), while non-synonymous SNPs in the coding region of this gene alters the ability of IL-33 to bind to its receptor (22). It is, therefore, possible that in those with asthma, one or more of these SNPs may be responsible for the translation of one splice variant over the other.

Jackson and colleagues recently demonstrated that RV-infected epithelial cells release IL-33 that induces peripheral blood naïve T-cells and ILC2 cells to produce type 2 cytokines under conditions of polyclonal activation (6). Our observations extend these findings to show that IL-33 can also enhance virus-specific type 2 cytokine release. We previously reported that RV-activated memory T-cells are responsible for much of the IFNγ and IL-13 production in healthy individuals (24). In contrast, the current study employed recently developed tools for accurately identifying human ILCs. We show herein that in those with asthma, ST2^+^ILC are in fact the main IL-13 producers, rather than conventional T-cells. ILC2 are commonly identified with either ST2 or the prostaglandin D2 receptor, CRTH2 (17). Therefore, we used CRTH2 to further characterize this population. As per Smith and colleagues (17), we found that a large proportion of these cells also express this marker (data not shown) and believe this population to be ILC2. Consistent with this observation, ILC2 have been shown to be the primary producers of IL-13 in the context of IL-33 in the asthmatic airway (25).

Although the different experimental stimuli induced only subtle changes in the frequency of cells producing type 1 and 2 cytokines (Figures 3 and 4) between asthmatic and healthy individuals, it seems likely that greater difference would have occurred at earlier time points, especially as we observed that IL-33 + RV induced a marked increase in secreted type 1 and 2 cytokines in cultured supernatant between asthmatic and non-asthmatics, respectively (Figure 1). Functional studies in mice show that IL-13^+^ ST2^+^ ILC2 are most prominent between 6 and 10 days post infection (26, 27). However, this varies between models. In humans, it is unclear when the frequency of IL-13^+^ILC2 peaks during RV Infection. In contrast, differences in IFNγ release in cultured supernatants between asthmatic and non-asthmatic donors can be detected as early as 48 h post infection (28). Therefore, greater differences in IFNγ^+^ cells in healthy individuals may be observed at this time point. Future studies should assess how ILC2, NK cells, and T-cells interact over time to amplify type 1 or type 2 cytokines in the context of RV infection. Our preliminary experiments indicated that numbers of ST2^+^ ILC and NK cells were similar in asthmatic and healthy participants (Figure S2 in Supplementary Material). However, it would be premature to read too much into these observations, which require confirmation in a much larger study.

Due to their substantial role in development of type 2 immune responses in allergic asthma, it is surprising that ST2^+^ILC have the ability to produce IFNγ in response to IL-33 in healthy individuals. Although, it has been suggested that ILC2 have a protective role *in vivo* (29), no clear link has been established between this subset and type 1 cytokine production. However, new evidence in humans has emerged that ILC2 from non-allergic donors express higher levels of *NKG7, SOC1*, and *TBX21*. These genes are known to suppress type 2 associated transcriptional programs (30). Furthermore, low *SOC1* gene expression in the airways of severe asthmatics has been shown to be inversely associated with airway eosinophilia (31).

Another novel finding to emerge from this study is that RV and IL-33 act together to augment NK cell IFNγ production. Although T cells are also thought to produce IFNγ subsequent experiments showed little evidence of T cell involvement (Figure S5 in Supplementary Material). Interestingly, a recent study has shown that IL-33 exposed NK cells have the ability to negatively regulate ILC2s in mice *via* an IFNγ-dependent mechanism (32). Although our study supports that of Bi et al. (32), the possibility also exists that ILC2s are regulated by another pathway that then supports more favorable conditions for IFNγ production. In line with this concept, IFNα production from plasmacytoid dendritic cells in the context of IL-33 has also been shown to suppress proliferation of ILC2 and alleviate airway hyperreactivity (33). Future studies should also assess the extent to which IFNα and IFNγ influence ILC2 proliferation during experimental RV infection in the context of IL-33. This would provide a greater understanding into the mechanisms that regulate ILC2.

There are a number of limitations of the study that must be acknowledged. We studied a population of mild to moderate allergic asthmatics, and future studies will need to examine if IL-33 has similar effects across a broader range of asthma phenotypes and degrees of asthma severity. Second, although people with asthma had a greater prevalence of allergic sensitization than non-asthmatic donors, our study was not large enough to determine if our findings are confounded by atopy. In addition, we do not know if the effects of IL-33 on circulating cells also holds true for resident lymphocytes within the airway, and this needs to be examined in future studies. We do believe, however, that our *in vitro* experiments provide insight into the likely effects of airway IL-33 on lymphocyte populations that have recently migrated into the airway mucosa, with responsiveness to IL-33 differing between lymphocytes from asthmatic and healthy individuals. Interestingly, findings from a large birth cohort study indicate that variations in RV-activated PBMC cytokine responses are associated with different clinical outcomes (3). Future studies should also directly examine interaction between lymphocyte populations and airway epithelial cells, and the extent to which this involves IL-33. Finally, there is evidence in asthma that T_reg_ cells are dysfunctional (34) and though we saw only minimal effects of IL-33 on T_reg_ (data not shown), our functional analysis was restricted to a small range of cytokines, and so this needs to be examined further.

In summary, these findings suggest that the effects of IL-33 on the cellular response to RV differ in asthmatic and healthy individuals, thus providing a potential mechanism by which RV infection in a high IL-33 tissue microenvironment can induce immunopathology in asthma but not in healthy people.

## Ethics Statement

The Metro South Human Research Ethics Committee approved this study (Ethics approval number 2008000037).

## Author Contributions

JU, LJ, YX, and ML contributed to the conception of the study; JU, LJ, YX, ML, and MC contributed to the design of the study; LJ and YX performed majority of the experiments and the statistical analysis. LJ wrote the manuscript; JU, YX, ML, MC, and LM contributed to the revision of this manuscript and approved the submitted version.

## Conflict of Interest Statement

The authors declare that the research was conducted in the absence of any commercial or financial relationships that could be construed as a potential conflict of interest.
