# Supplementary figures and images for: Interleukin 33 Selectively Augments Rhinovirus-Induced Type 2 Immune Responses in Asthmatic but not Healthy People
Source: Front Immunol. 2018 Aug 16;9:1895. doi: 10.3389/fimmu.2018.01895 (PMC6108046; doi:10.3389/fimmu.2018.01895)

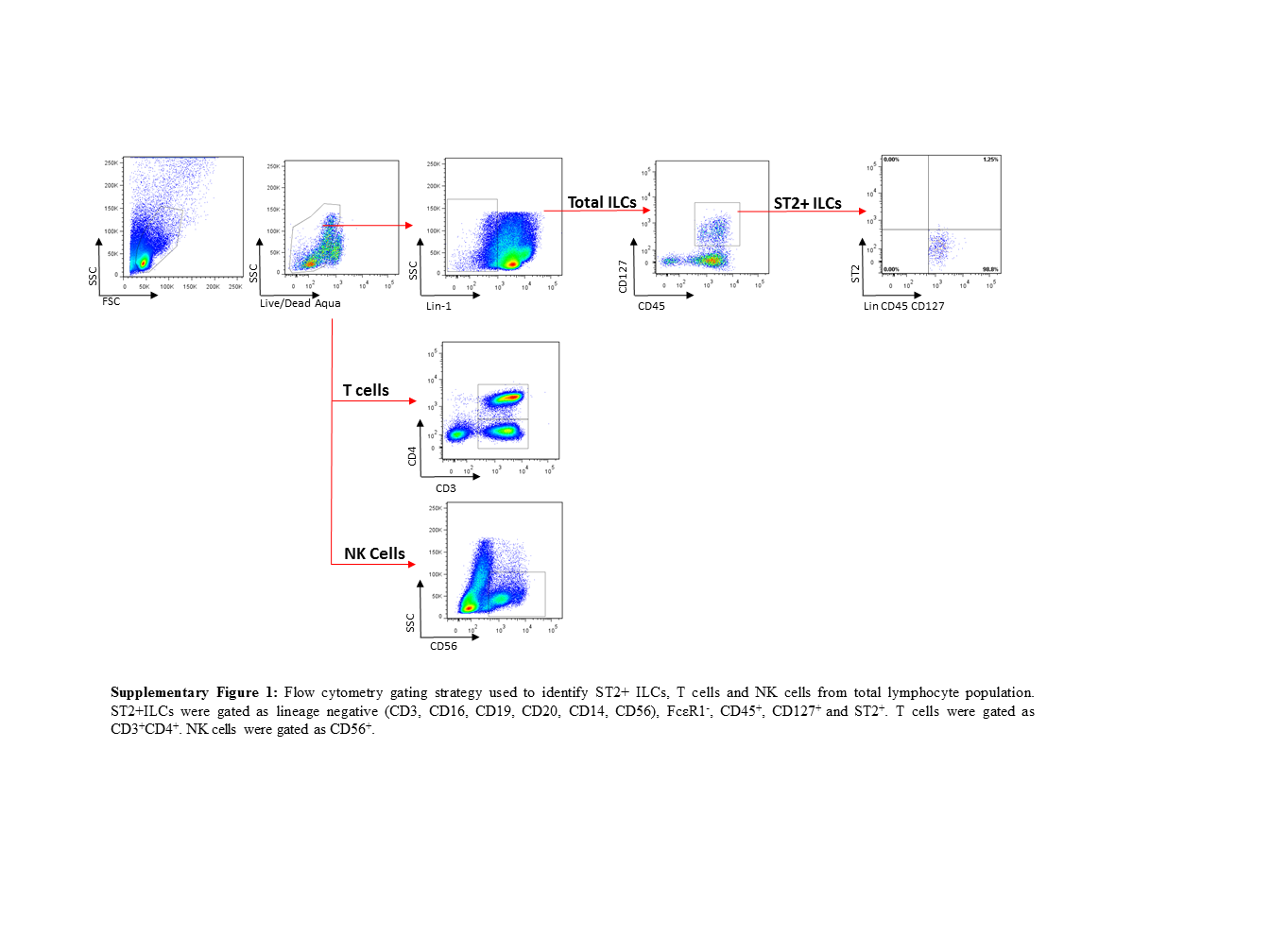

Supplement: Supplementary file 1 [file Image_1.tif]

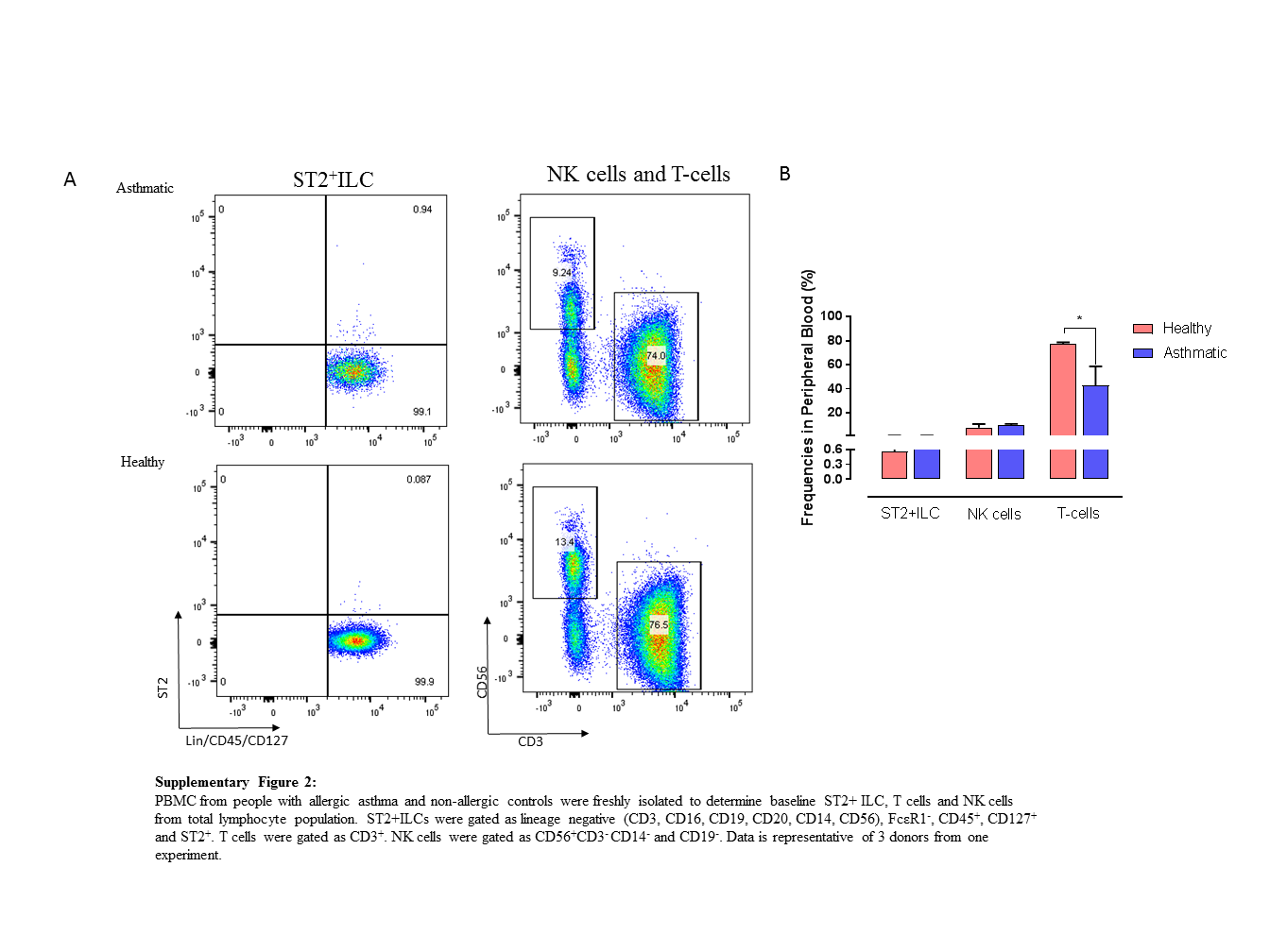

Supplement: Supplementary file 2 [file Image_2.tif]

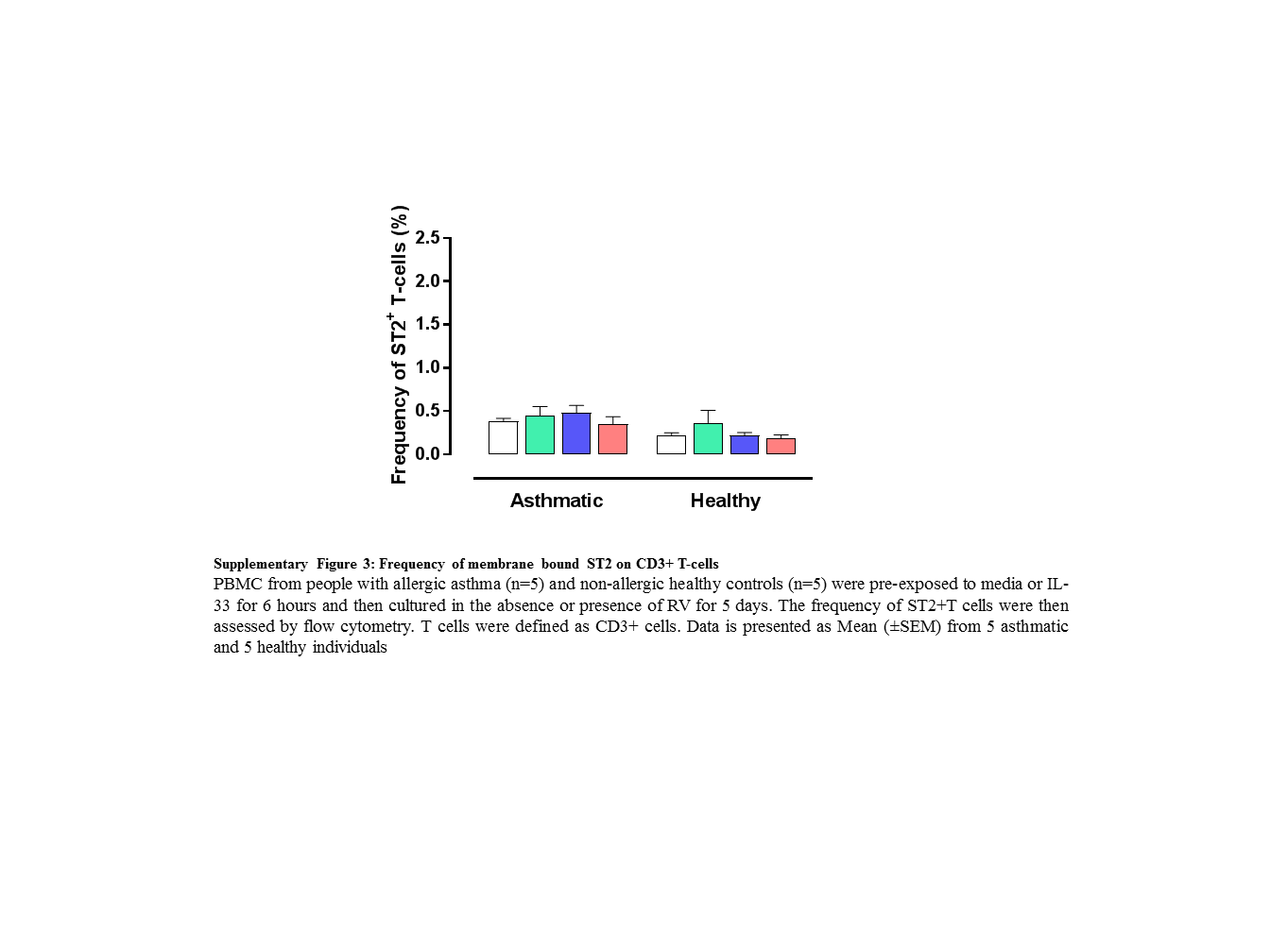

Supplement: Supplementary file 3 [file Image_3.tif]

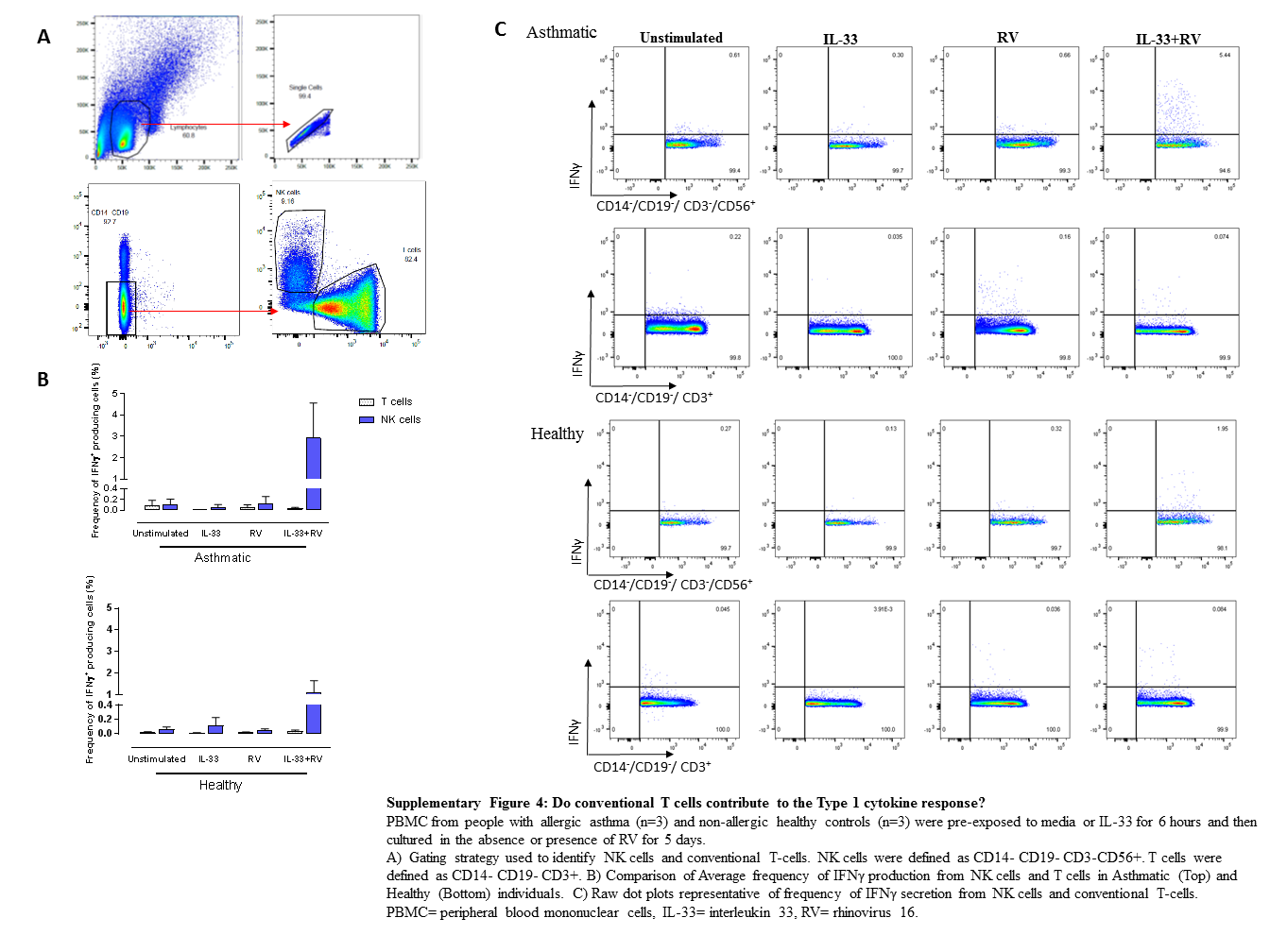

Supplement: Supplementary file 4 [file Image_4.tif]

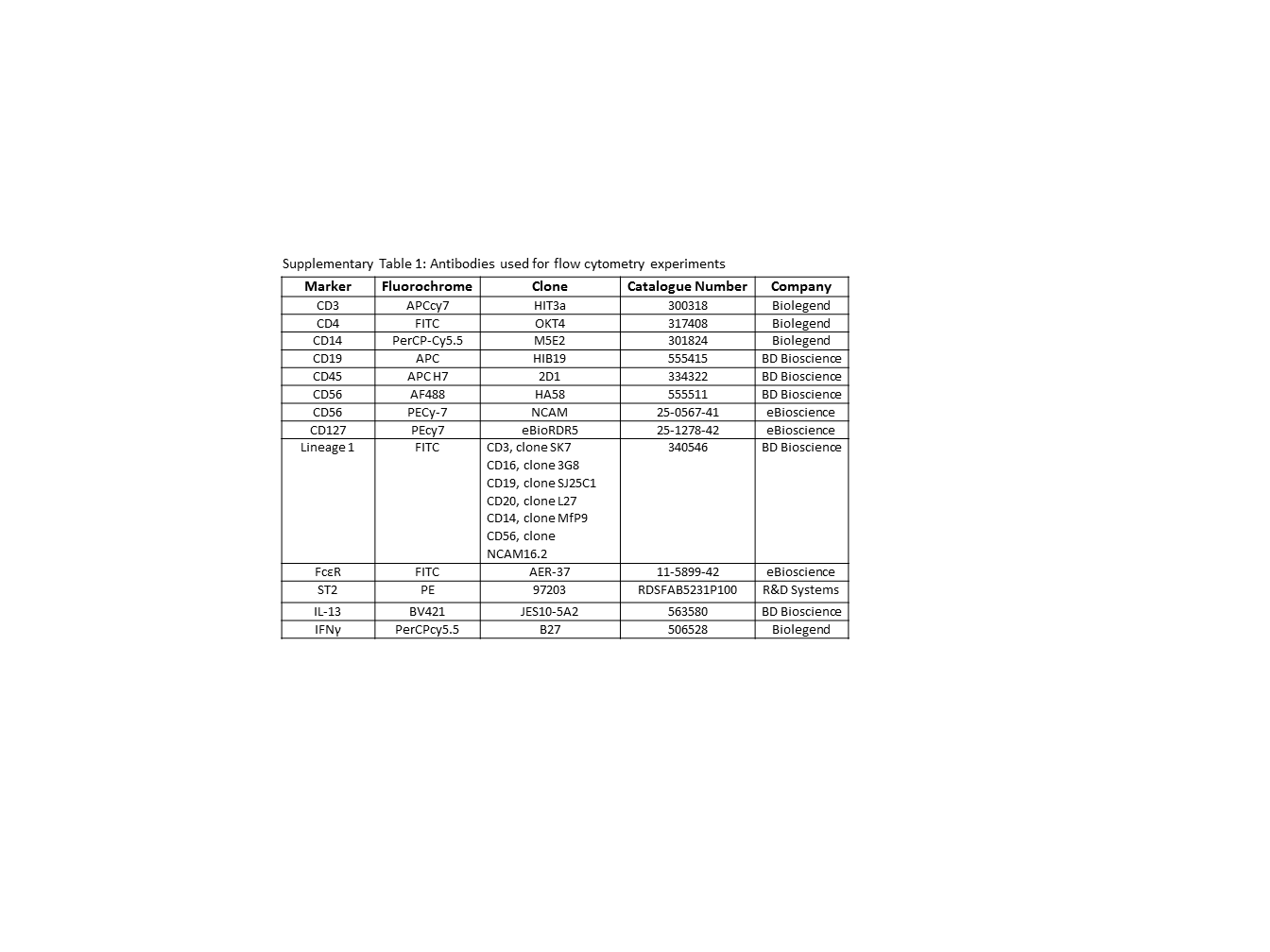

Supplement: Supplementary file 5 [file Image_5.tif]
